# Toc75‐V/OEP80 is processed during translocation into chloroplasts, and the membrane‐embedded form exposes its POTRA domain to the intermembrane space

**DOI:** 10.1002/2211-5463.12791

**Published:** 2020-02-17

**Authors:** Lucia E. Gross, Nicole Spies, Stefan Simm, Enrico Schleiff

**Affiliations:** ^1^ Department of Molecular Cell Biology of Plants Goethe University Frankfurt Germany; ^2^ Frankfurt Institute for Advanced Studies Germany; ^3^ Buchmann Institute for Molecular Life Sciences Goethe University Frankfurt Germany

**Keywords:** chloroplasts, OEP80, protein import, protein membrane insertion, Toc75‐V, β‐barrel membrane protein

## Abstract

The insertion of membrane proteins requires proteinaceous complexes in the cytoplasm, the membrane, and the lumen of organelles. Most of the required complexes have been described, while the components for insertion of β‐barrel‐type proteins into the outer membrane of chloroplasts remain unknown. The same holds true for the signals required for the insertion of β‐barrel‐type proteins. At present, only the processing of Toc75‐III, the β‐barrel‐type protein of the central chloroplast translocon with an atypical signal, has been explored in detail. However, it has been debated whether Toc75‐V/ outer envelope protein 80 (OEP80), a second protein of the same family, contains a signal and undergoes processing. To substantiate the hypothesis that Toc75‐V/OEP80 is processed as well, we reinvestigated the processing in a protoplast‐based assay as well as in native membranes. Our results confirm the existence of a cleavable segment. By protease protection and pegylation, we observed intermembrane space localization of the soluble N‐terminal domain. Thus, Toc75‐V contains a cleavable N‐terminal signal and exposes its polypeptide transport‐associated domains to the intermembrane space of plastids, where it likely interacts with its substrates.

## Graphical abstract

Toc75‐V is a β‐barrel protein with N‐terminal soluble polypeptide transport‐associated (POTRA) domains. The protein is synthesized in the cytosol with an N‐terminal signal for targeting to chloroplasts, which is processed at a conserved cleavage site (shown for the pea protein) after translocation across the translocon of the outer envelope of chloroplast machinery. The membrane‐inserted Toc75‐V exposes its POTRA domains for substrate recognition to the intermembrane space.

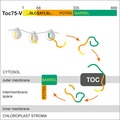

**Abbreviations**
OEP: outer envelope protein
OEV: chloroplast outer envelope membrane vesicles
POTRA: polypeptide transport‐associated
TIC: translocon of the inner envelope of chloroplast
TOC: translocon of the outer envelope of chloroplast

Protein translocation across and protein insertion into membranes is central for cellular function 1, 2, 3, 4. Translocation of most plastidic proteins across the outer membrane of the organelle is facilitated by the translocon on the outer envelope membrane of chloroplasts—TOC 2, 5, 6. The TOC core complex is composed of Toc34, Toc75, and Toc159. Each of these components comprises a protein family, where the different proteins often but not always have overlapping functions 7. The central component of the TOC translocon is Toc75, which forms a β‐barrel‐type protein translocation channel and contains three N‐terminal soluble polypeptide transport‐associated (POTRA) domains 8, 9. After the genome of *Arabidopsis thaliana* became available, three genes were identified to code for members of the Toc75 protein family involved in the precursor protein translocation across membranes annotated as Toc75‐I, Toc75‐III, and Toc75‐IV 7. Subsequent studies demonstrated that *Toc75‐I* is a pseudogene, *Toc75‐IV* might play a role for growth in the dark, and *Toc75‐III* codes for the central translocation pore 10. Utilizing proteomics, an additional protein of this family was discovered and annotated as Toc75‐V/outer envelope protein 80 (Oep80; Toc75‐V hereafter) 11. The protein contains three POTRA domains as well, which are expected to be localized in the intermembrane space 12. Like Toc75‐III 10, Toc75‐V is essential for plant development 13, 14, 15. Two additional genes with similarity to Toc75‐V have been identified as well, namely P39 and P36 16, 17, 18, 19, 20. Interestingly, these two proteins do not contain N‐terminal POTRA domains and mutants of these two genes are viable 16, 18. Moreover, P39 and P36 originated from a recent genome duplication of *A. thaliana* and only one gene is found in most of the other plant species examined 17. A recent study revealed that P39 (SP2) might be the conducting component of a retro‐translocon, which seems to facilitate the extraction of ubiquitinated TOC proteins from the outer membrane for proteasomal degradation in the cytosol 21.

Reconstruction of the phylogenetic relation between the Toc75‐III group, the Toc75‐V group, and the bacterial ancestors revealed that the Toc75‐V group is more closely related to bacterial proteins 19, 20. The divergence between the two groups of plant proteins can be attributed to different amino acid signatures of the barrel region of the Toc75‐III and Toc75‐V proteins 16, 20. In addition, Toc75‐III and Toc75‐V exhibit a different targeting signature. Toc75‐III has a bipartite targeting signal 22 that contains a poly‐glycine stretch 23. The signal is cleaved by the stromal 8 and the intermembrane space localized type I signal peptidase 24. In contrast, Toc75‐V does not contain such a bipartite signal and even the presence of a cleavable N‐terminal transit peptide is under debate 11, 12, 13, 15, 25, 26, 27. Initially, it was concluded that Toc75‐V in *A. thaliana* does not contain a cleavable signal. This judgment was based on *in vitro* import experiments and immunodecoration of isolated chloroplasts 25. In a subsequent study, the migration at 80 kDa leading to renaming the protein to OEP80 was disputed by the same authors. Using another antibody raised against Toc75‐V, they observed a migration of the protein in endogenous membranes at 70 kDa 15, which was comparable to the molecular weight observed for the protein in *Pisum sativum* 11. Again, the same authors challenged the *in vitro* import results as well and suggested the presence of a cleavable signal 12. Finally, a recent approach by these authors using *in vitro* import and stromal processing assay provided further support for the existence of a cleavable signal 27. Before, mutant versions of Toc75‐V were generated utilizing the second ATG as start codon. The results were interpreted as such that either Toc75‐V is translated by two alternative start codons or by the presence of a cleavable signal 13.

To provide independent evidence in this discussion, we reinvestigated the presence of a cleavable N‐terminal portion and the topology of Toc75‐V. We confirm the suggested existence of a cleavable N terminus by *in vivo* import experiments and by N‐terminal sequencing of the protein in endogenous membranes. Moreover, we confirm that the soluble POTRA domains of Toc75‐V are oriented toward the intermembrane space, which strengthens the current topology model derived by protease protection 11 and import experiments 27. The implications for possible structural models are discussed.

## Materials and methods

### Bioinformatics analyses

Orthologues and co‐orthologues for Toc75‐V were acquired by reciprocal best‐BLAST hit search between *A. thaliana* proteome (TAIR10) and *P. sativum* proteome (UniProt) using NCBI BLAST. Sequence alignments of amino acid sequences of Toc75‐V from *P. sativum*, *Medicago truncatula*, *Glycine max*, *Solanum lycopersicum*, *Brassica rapa*, *A. thaliana*, and *Chlamydomonas reinhardtii* were performed using clustal omega 28 and mafft 29. Secondary structure prediction for β‐barrel proteins was performed as previously established 30, 31, 32.

### Isolation of total RNA and generation of cDNA

*Arabidopsis thaliana* total RNA was purified using the E.Z.N.A.® Plant RNA Kit (Omega Bio‐Tek, Norcross, GA, USA) according to the manufacturer’s recommendation. *Pisum sativum* RNA was isolated by homogenizing leaves from 5‐day‐old seedlings in liquid nitrogen. One hundred milligram material was resuspended in 1 mL of 0.4 m ammonium thiocyanate, 0.8 m guanidinium thiocyanate, 0.1 m sodium acetate, 5% (v/v) glycerol, 40 % (w/v) phenol, pH 5. After centrifugation (10 min, 12 000 ***g***, 4 °C), the supernatant was supplemented with 0.2 mL chloroform, vortexed, and incubated for 10 min at RT. After centrifugation (15 min, 12 000 ***g***, 4 °C), the RNA in the aqueous phase was precipitated by addition of 125 µL isopropanol and 125 µL of 1.2 m NaCl, 0.5 m sodium citrate in DEPC‐treated H_2_O. After centrifugation (12 000 ***g***, 10 min, 4 °C), the pellets were washed with 70% ethanol, dried, and resuspended in DEPC‐treated H_2_O. Residual DNA was removed by addition of RNase‐free DNase (E.Z.N.A.® Plant RNA Kit; Omega Bio‐Tek) for 3 h at 37 °C in the recommended buffer supplemented with 40 U RiboLock RNase Inhibitor (Thermo Fisher Scientific, Darmstadt, Germany). The DNAse was inactivated (8 mm EDTA, 75 °C, 10 min), and RNA was precipitated overnight with 2.5 volumes of 95% ethanol. After centrifugation and washing, the RNA was resuspended in DEPC‐treated H_2_O. cDNA was generated by reverse transcription using the RevertAid Reverse Transcriptase (Thermo Fisher Scientific) according to the recommendations. RNA sequencing and assembly using BBmap 33 is described in the legend of Fig. S1.

### Constructs

Oligonucleotides are listed in Table S1 and constructs in Table S2 34, 35, 36. psToc75‐V was amplified by PCR using primers annealing at 5′ or 3′ UTR derived from RNASeq. The product was introduced in pSP65 (Promega, Walldorf, Germany; SacI/PstI) and sequenced. The CDS was amplified from cDNA, cloned into pSP65 by Gibson assembly 35, and sequenced. A 2× tandem FLAG tag was amplified and cloned into pAVA_11N or pAVA_11C (NcoI/XbaI) 37, respectively. The NcoI site was deleted from pAVA_C‐FLAG by plasmid linearization with NcoI, mung bean nuclease treatment (New England BioLabs, Frankfurt/Main, Germany), and blunt‐end ligation. atToc75‐V from pAVA_11N 37 was cloned into these FLAG‐containing vectors using Acc65I/SpeI. Methionine substitutions and LCCA (aa: 123–126) deletion were introduced by site‐directed mutagenesis PCR (QuikChange mutagenesis kit; Stratagene, La Jolla, CA, USA). Silent point mutations were introduced to generate restriction sites for identification of positive clones. POTRA domains of atToc75‐V were amplified from existing constructs and cloned into pET24c (NdeI/NotI). Toc75‐V with N‐terminal MBP‐tag and C‐terminal 6xHIS‐tag was created by amplification of POTRA domains and cloning into pMal (NEB; BamHI/NotI).

### Purification of recombinant proteins and antibody generation

6xHIS‐tagged atToc75‐V (amino acids 154–396; Toc75‐V_P1‐3) were produced in *Escherichia coli* after addition of 1 mm IPTG and expression for 3 h at 37 °C. Cells were suspended in 50 mm Tris/HCl pH 8, lysed by French pressing, centrifuged, and pellets washed once with wash buffer [50 mm Tris/HCl pH 8, 1 m urea, 1% (v/v) Triton X‐100] and once with wash buffer lacking detergent. Inclusion bodies were solubilized in 50 mm Tris/HCl, 300 mm NaCl, and 8 m urea (overnight, RT). Toc75‐V_P1‐3_his was subjected to Ni‐NTA. The matrix was washed with 50 mm Tris/HCl, 500 mm NaCl, 6 m urea, and 10 mm imidazole and the protein eluted by addition of 50 mm Tris/HCl, 250 mm NaCl, 6 m urea, and 500 mm imidazole.

For antibody production, the protein was subjected to SDS/PAGE, was reversibly stained by 100 mm KCl, and directly excised. Two rabbits were immunized (Pineda Antikörper‐Service, Berlin, Germany). Other antibodies were described (Table S3) 37, 38, 39. For antibody purification, soluble MBP_Toc75‐V_P1‐3_his was expressed at 23 °C for 3 h in *E. coli* BL21 cells. After harvesting, cells were resuspended in 50 mm HEPES/KOH pH 8, 20% (v/v) glycerol, 250 mm NaCl, and 5 mm MgCl_2_ including 2 mg·mL^−1^ lysozyme. Cells were lysed by sonication, and cell debris was removed by centrifugation (20 000 ***g***, 30 min, 4 °C). The supernatant was subjected to Ni‐NTA purification. After washing the matrix with 10 × 10 cv of 50 mm HEPES/KOH pH 8, 20% (v/v) glycerol, 5 mm MgCl_2_, 500 mm NaCl, and 20 mm imidazole, proteins were eluted in 50 mm HEPES/KOH pH 8, 20% (v/v) glycerol, 250 mm NaCl, 5 mm MgCl_2_, and 500 mm imidazole.

Antibodies were purified as follows: Purified MBP_Toc75‐V_P1‐3_his was subjected to buffer exchange via PD‐10 desalting columns (GE Healthcare, Frankfurt, Germany) to 0.1 m NaHCO_3_ pH 8.3, 0.5 m NaCl. The protein was coupled to CNBr‐activated SepharoseTM 4B (GE Healthcare) at 4 °C overnight under constant rotation. Excess protein was removed by washing the column with five column volumes of coupling buffer. Reactive groups of the CNBr Sepharose were blocked by incubation with 1 m ethanolamine pH 8 for 2 h at RT. The matrix was washed by three cycles of alternating steps of 0.1 m Na/acetate pH 4, 0.5 m NaCl and 0.1 m Tris/HCl pH 8, 0.5 m NaCl. Antibodies were purified from serum by incubation with MBP_Toc75‐V_his coupled matrix at 4 °C overnight under constant rotation. The column was washed with PBS buffer, and bound antibodies were eluted with 0.2 m glycine pH 2.2 into a tube containing 2 m Tris/HCl pH 8.8. The purified antibodies were precipitated by addition of saturated ammonium sulfate, pelleted (20 000 ***g***, 30 min, 4 °C), and resuspended in PBS buffer.

### Protein extraction, organelle and envelope isolation, and treatments

Total protein of leaf tissue from *A. thaliana* or *P. sativum* was extracted by homogenization in liquid nitrogen. The material was resuspended in 40 mm Tris/HCl, pH 6.8, 8 m urea, 0.1 mm EDTA, 5% (w/v) SDS, 2% (v/v) β‐mercaptoethanol, and 0.04% (w/v) bromophenol blue and subjected to a clearing centrifugation. Chloroplasts and chloroplast outer envelope vesicles from *P. sativum* were isolated as in Ref. 38.

Treatments were adopted from previously established protocols 39, 40. In brief, thermolysin treatment of organelles was performed by resuspending chloroplasts (20 µg chlorophyll) in 100 µL of 50 mm HEPES/KOH pH 7.6, 330 mm sorbitol, 1 mm CaCl_2_, and thermolysin (Calbiochem) at a final concentration of 240 µg·mL^−1^ (30 min, ice, dark). Proteolysis reaction was quenched by addition of 5 mm EDTA. Organelles were harvested at 1500 ***g*** for 2 min and washed with 50 mm HEPES/KOH pH 7.6 and 330 mm sorbitol. Thermolysin treatment under hypo‐osmotic conditions was performed as described above in 50 mm HEPES/KOH pH 7.6. Organelles after thermolysin during osmolysis treatment were harvested by centrifugation (100 000 ***g***, 30 min, 4 °C) and washed once. Chloroplasts were resuspended in SDS loading buffer, and organelles equal to 10 µg were subjected to SDS/PAGE and western blotting.

### Pegylation

Pegylation was performed as described 41. In brief, prior to pegylation, the chloroplasts were incubated in 100 mm Tris/HCl pH 7 and 330 mm sorbitol in the presence (Fig. 3D) or absence of 10 mm β‐mercaptoethanol (5 min, ice, dark, Fig. 3E). After washing in buffer lacking the reducing agent, the control was incubated with 100 mm Tris/HCl pH 7, 330 mm sorbitol, and 1 mm EDTA. For pegylation, the buffer was supplemented with 10 mm Peg‐Mal under iso‐osmotic conditions. Peg‐Mal crosslinking during osmolysis was performed in the same buffer lacking sorbitol (20 min, ice, dark). Peg‐Mal crosslinking during SDS‐based solubilization was performed in the buffer with sorbitol and additional 2% SDS (20 min, ice, dark). The reaction was quenched by addition of DTT (100 mm final). The intact chloroplasts were harvested (1500 ***g***, 1 min), while total membranes of osmolytically lysed chloroplasts were centrifuged at 100 000 ***g***, 30 min, 4 °C. All samples were resuspended in SDS loading buffer and subjected to SDS/PAGE and western blotting. For the samples shown in Fig. 3E, acetone precipitation was used to remove the excess of uncrosslinked Peg‐Mal 42.

### Transformation of *A. thaliana* and *S. lycopersicum* mesophyll protoplasts

Mesophyll protoplasts from *A. thaliana* were isolated and transformed according to established protocols 37, 43. For PEG facilitated transformation of protoplasts, an amount of 5–10 µg (*A. thaliana*) or 20 µg (*S. lycopersicum*) plasmid DNA for 10^5^ cells was used. Expression of the proteins was performed overnight. Where indicated, osmolysis of protoplasts was performed by incubation with 50 mm HEPES/KOH, pH 7.6, carbonate treatment by incubation with 0.1 m Na_2_CO_3_, and detergent solubilization with 50 mm HEPES/KOH, pH 7.6 supplemented with 1% Triton X‐100. All samples contained 1% (v/v) Protease Inhibitor Cocktail for plant cell and tissue extract (PIC; Merck, Darmstadt, Germany). The samples were incubated for 30 min on ice and separated into pellet and supernatant by centrifugation at 100 000 ***g*** for 30 min, 4 °C. The proteins were denatured with SDS loading buffer.

### Immunoprecipitation experiments

Purified αToc75‐V antibodies were immobilized on 0.1 m sodium phosphate pH 8 pre‐equilibrated Protein A Sepharose 4 fast flow (GE Healthcare) as described in protocols (NEB BioLabs). After removal of the supernatant, beads were washed thrice with 0.1 m sodium phosphate pH 8 and twice with 0.2 m triethanolamine pH 8.2. The crosslinking reaction was initiated by addition of 6.5 mg·mL^−1^ DMP in 0.2 m triethanolamine pH 8.2 (45 min, RT). After sedimentation of beads, the supernatant was removed and crosslinking was quenched by incubation with 0.1 m ethanolamine (1 h, RT). The quenching solution was removed, and beads were washed with PBS. Uncrosslinked antibody was eluted with 0.1 m glycine pH 2.5. Subsequently, the beads were thoroughly washed with PBS and stored at 4 °C. For Edman degradation, 500 µg of total chloroplast outer envelope membrane vesicles (OEV) protein was denatured in 1% SDS and 0.5% TX in PBS buffer (5 min, 25 °C). After diluting the sample 10‐fold in PBS containing 0.5% TX and centrifugation (20 000 ***g***, 15 min), the supernatant was incubated with αToc75‐V crosslinked to Protein A Sepharose (2 h, RT). Beads were washed three times with 1 mL PBS containing 0.5% TX. Bound proteins were eluted in alternating steps of 0.3 m glycine pH 2.2 and PBS. After SDS/PAGE analyses of 10% of the elutions, the remaining samples were pooled, and trichloroacetic acid was precipitated and subjected to SDS/PAGE and transferred onto a PVDF membrane. The proteins were visualized by staining with 0.1% CBB R250, 40% methanol, and 10% acetic acid. Edman degradation was performed by Proteome Factory AG (Berlin, Germany).

## Results

### Toc75‐V is processed upon transport into chloroplasts

**Figure 1:**
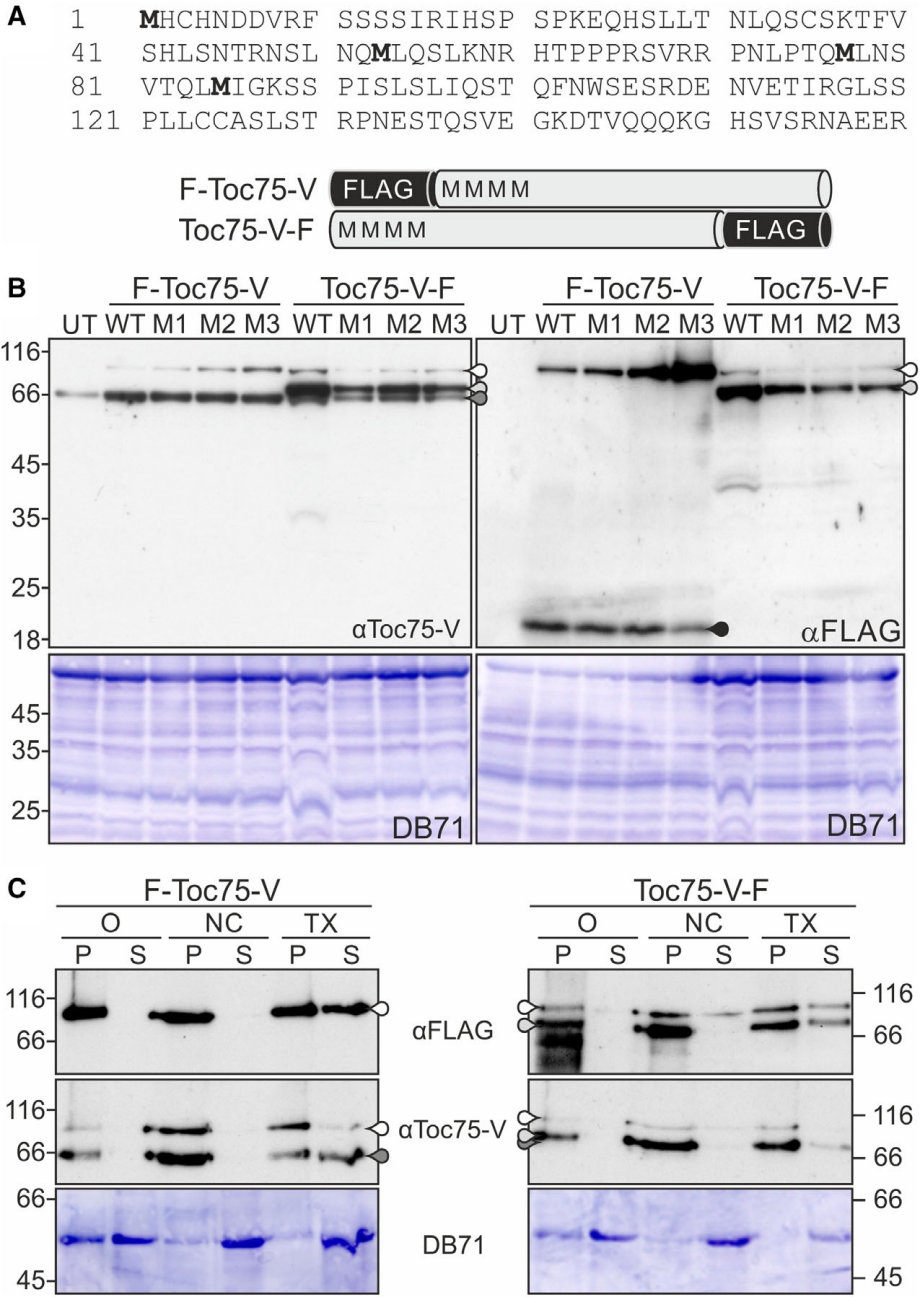
Processing of FLAG‐tagged Toc75‐V. (A) The amino acid sequence of the N terminus of atToc75‐V and schemes of the constructs are shown. (B) *Arabidopsis thaliana* protoplasts expressing different versions of FLAG‐tagged atToc75‐V (WT; M53A, M1; M53A/M77A, M2; M53A/M77A/M85A, M3) were harvested and the proteins subjected to western blotting with αToc75‐V (left) and αFLAG antibodies (right). White droplets: migration of unprocessed FLAG‐tagged proteins, gray droplets: processed protein with C‐terminal FLAG, dark gray droplet: processed proteins of FLAG‐Toc75‐V and of endogenous Toc75‐V, black droplet: a FLAG antibody reactive band, which might represent the N‐terminal cleaved fragment as discussed in the text. UT, untransformed protoplasts. On the bottom, a section of the DB71 stain is shown for loading control. (C) *Arabidopsis thaliana* protoplasts transformed with FLAG‐atToc75‐V or atToc75‐V‐FLAG were lysed after 14 h in hypo‐osmotic buffer (O) and incubated with 100 mm Na_2_CO_3_ (NC) or 1.0% Triton X‐100 (TX). Equal amounts of pellet (P) and supernatant (S) were subjected to two SDS/PAGEs followed by western blotting with either αFLAG (upper panels) or αToc75‐V antibodies (middle panels). Droplets as in (B). The DB71 staining of RuBisCO (lower panels) of the blot subsequently incubated with αToc75‐V antibodies is shown for loading control and confirmation of the treatment efficiency.

It was speculated that Toc75‐V from *A. thaliana* (atToc75‐V) contains a cleavable N‐terminal sequence 11, 13, although the N‐terminal 52 amino acids are not essential for targeting 15 We analyzed the processing of atToc75‐V by generating N‐ or C‐terminal FLAG‐tagged atToc75‐V constructs (Fig. 1A). After their expression in *A. thaliana* protoplasts and immunostaining with antibodies against the atToc75‐V‐POTRA domains (αToc75‐V), it became apparent that both fusion proteins migrated in two distinct forms (Fig. 1B). The molecular weight of one form corresponded to the full‐length proteins including the tag (Fig. 1B, αToc75‐V, white droplet), the other to the endogenous atToc75‐V in case of FLAG‐atToc75‐V (F‐Toc75‐V, Fig. 1B, αToc75‐V, dark gray droplet) or slightly higher consistent with the presence of the C‐terminal tag when using atToc75‐V‐FLAG (Toc75‐V‐F, Fig. 1B, αToc75‐V, light gray droplet). After expression of FLAG‐atToc75‐V, the tag was only detected in the band of higher molecular weight (Fig. 1B, αFLAG, white droplet), while both forms of the C‐terminal fusion protein contained the tag (Fig. 1B, αFLAG, white and light gray droplets). Moreover, expression of FLAG‐atToc75‐V resulted in the occurrence of a small‐molecular‐weight fragment of about 18 kDa, which was detectable by αFLAG antibodies (Fig. 1B, black droplet). This might represent the cleaved N‐terminal fragment. Methionine 53 (M1), methionines 53 and 77 (M2), and methionines 53, 77, and 85 (M3) were replaced by alanine (Fig. 1A), and expression of these constructs in protoplasts revealed an indistinguishable migration behavior when compared to wild‐type (WT) FLAG‐tagged atToc75‐V (Fig. 1B). Thus, the two forms resulted from processing and not from translation from a downstream start codon. The processed forms of FLAG‐atToc75‐V and atToc75‐V‐FLAG were membrane integral, as the proteins remained in the pellet after treatment with 100 mm Na_2_CO_3_ (Fig. 1C, NC/P). In turn, they became partially soluble after treatment with Triton X‐100 (Fig. 1C, TX/S). This points toward an N‐terminal processing of atToc75‐V during or after protein insertion. Worth mentioning, the unprocessed form of FLAG‐atToc75‐V and in parts of atToc75‐V‐FLAG remained in the pellet after carbonate extraction (Fig. 1C, NC/P). However, this protein became only partially soluble after treatment with detergent suggesting that the excess of atToc75‐V remaining as unprocessed protein was largely aggregated in the cell.

### Toc75‐V processing site is conserved

**Figure 2:**
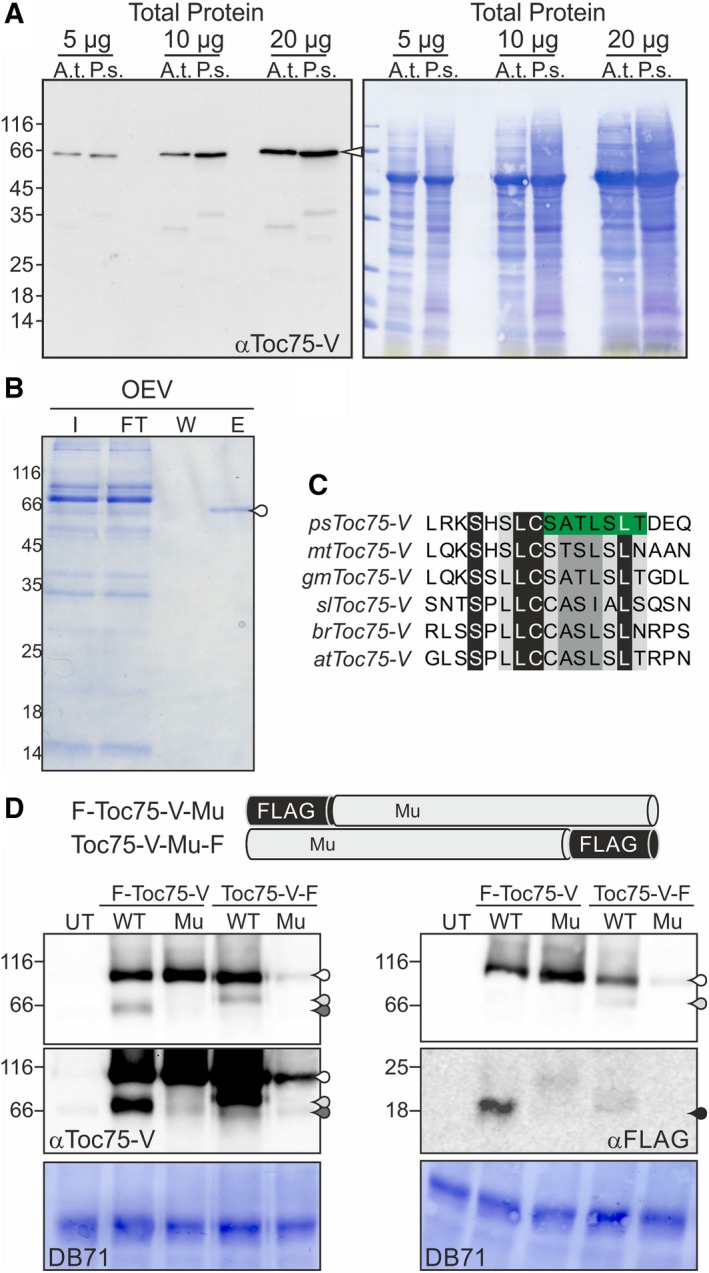
Identification of the N terminus of Toc75‐V occurring in native membranes. (A) Indicated amounts of total protein of *Arabidopsis thaliana* or *P. sativum* lysate were loaded on SDS/PAGE and subjected to western blotting. The membrane was stained with DB71 (right) and incubated with antibodies against αToc75‐V. This blot indicates that both endogenous proteins migrate with comparable molecular weight at about 66 kDa. (B) Solubilized *P. sativum* OEVs were subjected to immunoprecipitation using αToc75‐V‐Protein A Sepharose. 0.4% of OEVs (I) and flow‐through (FT), 10% of last wash (W), and first elution (E) fraction were subjected to SDS/PAGE followed by Coomassie Blue staining. The indicated band was extracted from all elution fractions and subjected to Edman degradation. (C) Amino acid sequence alignment of Toc75‐V from *P. sativum, Medicago truncatula, Glycine max, Solanum lycopersicum, Brassica rapa*, and *A. thaliana* created as described and visualized with Jalview (53, Fig. S2). Highlighted in green is the region of the mature start of psToc75‐V. (D) *Solanum lycopersicum* protoplasts expressing different versions of atToc75‐V with N‐terminal (F‐Toc75‐V) or C‐terminal (Toc75‐V‐F) FLAG tag (WT; with deletion of the LCCA motif: Mu, aa 123–126) were harvested, and the proteins were subjected to western blotting with αToc75‐V (top left) and αFLAG antibodies (top right). The contrast of the middle panels was modified to ensure visibility of the endogenous Toc75‐V in *S. lycopersicum* protoplasts (middle left) and to highlight the shorter FLAG‐containing fragment (middle right). On the bottom, the DB71 is shown as loading control. Labeling is taken from Fig. 1B. UT, untransformed protoplasts.

We analyzed the processing of Toc75‐V from *P. sativum* (psToc75‐V) to generalize the observation, as both atToc75‐V and psToc75‐V migrated at a similar molecular weight (Fig. 2A). We isolated RNA from 5‐day‐old pea seedlings followed by RNA sequencing and mRNA assembly. We identified the full‐length CDS of psTOC75‐V by amplification via PCR and sequencing of independent clones (Fig. S1). Comparison of the amino acid sequence with Toc75‐V sequences of *A. thaliana* and *M. truncatula* (Fig. S2) revealed an insertion in atToc75‐V at the N terminus upstream of the proposed POTRA domains. N‐terminal sequencing of immunoprecipitated endogenous psToc75‐V (Fig. 2B) by Edman degradation led to the identification of a peptide matching with a region starting about 30 amino acids upstream of the POTRA domains beginning at serine 68 (Fig. S1, 2). The identified N terminus represents a motif conserved in Toc75‐V in general (Fig. 2C). Thus, it is likely that atToc75‐V is processed at the same site.

To confirm the importance and conservation of the conserved region for processing in atToc75‐V, we introduced a deletion of the LCCA motif in the FLAG‐tagged atToc75‐V proteins (aa: 123–126), which correspond to the amino acids surrounding the Edman‐derived processing site of psToc75‐V (LC‐SA; Fig. 2C, 2). Subsequently, the WT proteins and the proteins carrying the deletion (Toc75‐V‐Mu‐F; F‐Toc75‐V‐Mu) were expressed in protoplasts, which were analyzed as described (e.g., Fig. 1B). Analysis of the processing pattern by immunodecoration with antibodies against Toc75‐V showed the processing of the WT proteins, but not of the proteins with deletion of the LCCA motif (Fig. 2D, top panel left). The endogenous protein became visible after overexposure of the western blot (Fig. 2D, middle panel left, white droplet). Using the FLAG tag detecting antibodies, we detected the processed form of the WT protein with the C‐terminal FLAG tag (Fig. 2D, top panel right, light gray droplet), but as before not of the proteins with N‐terminal FLAG tags or of the deletion mutant (Fig. 2D, top panel right). The small fragment that has been observed previously (Fig. 1B, black droplet) and might represent the cleaved off signal became only detectable after enhanced exposure for the WT protein with N‐terminal FLAG tag (Fig. 2D, middle panel right). Remarkably, the level of the mutant with C‐terminal FLAG tag was reproducibly lower than the level of the other proteins (Fig. 2D, top panel right, white droplet). However, as the protein was still detectable, we can demonstrate that this construct was not processed. Thus, we conclude that the four amino acids 123–126 are essential for the processing of Toc75‐V.

### Toc75‐V exposes the POTRA domain to the intermembrane space

**Figure 3:**
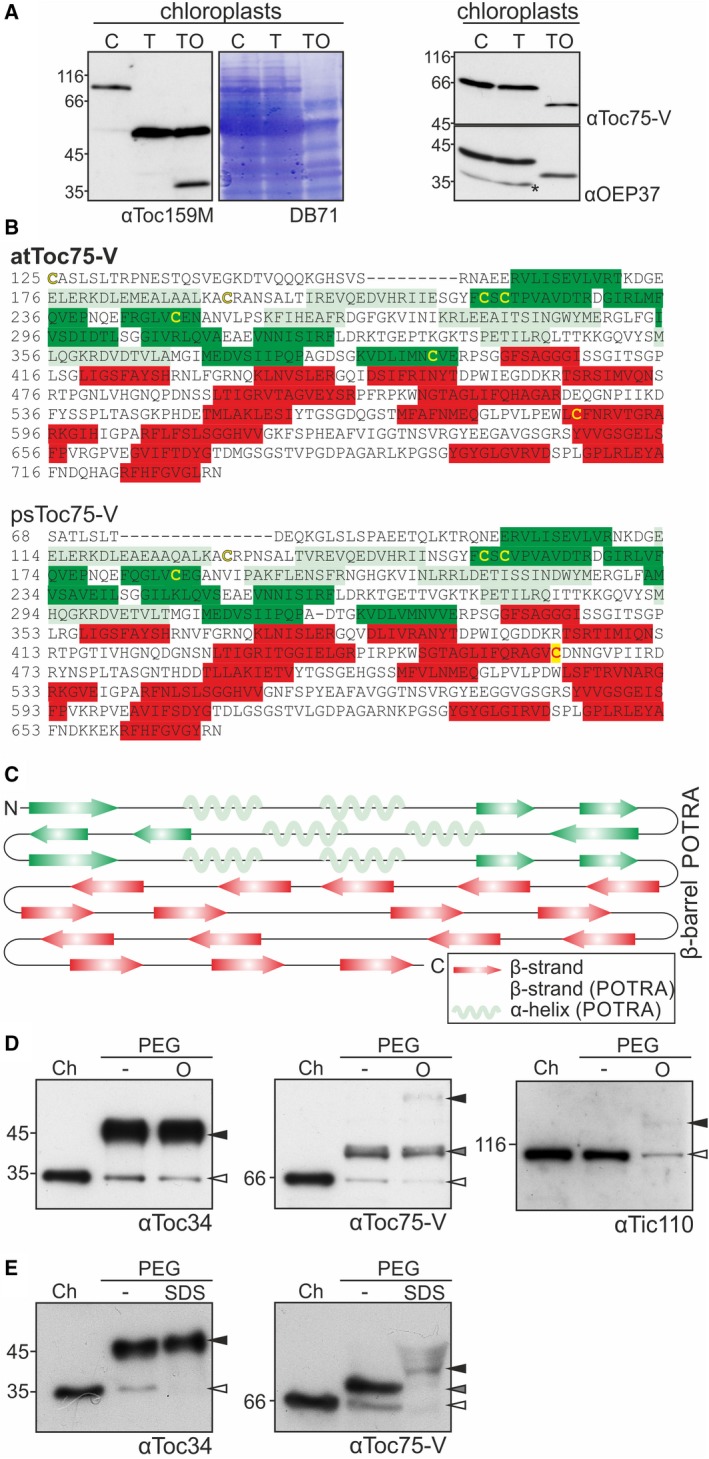
The localization of the N terminus of Toc75‐V in the intermembrane space. (A) *Pisum sativum* chloroplasts remained untreated (10 µg chlorophyll; C) were incubated with thermolysin under iso‐osmotic conditions (T) or during osmotic lysis (TO) followed by protein separation and western blotting with the indicated antibodies. Star: cross‐reactivity of subsequent immunodecoration with multiple antibodies. The DB71 stain is shown for loading comparison. (B) Sequence of the mature domains of psToc75‐V and atToc75‐V after alignment is shown. The colored letters show the secondary structure prediction based on the consensus of the meta‐server Genesilico. Red highlighted letters represent β‐strands, and green highlighted letters represent POTRA domain secondary structures (light…helix; dark…strand). Cysteine residues are indicated as yellow letters. (C) The consensus secondary structure of the mature domain of Toc75‐V based on *A. thaliana* and *P. sativum* is drawn. Arrows represent β‐strands (red…β‐barrel; green…POTRA), and wavy lines represent α‐helices. (D) *Pisum sativum* chloroplasts remained untreated (Ch) were incubated with 10 mm PEG‐maleimide under iso‐osmotic conditions (−) or during lysis of chloroplasts (O). The reaction was stopped with 100 mm DTT; organelles were harvested and subjected to western blotting. White arrows: unmodified proteins, black: bands with the highest pegylation grade observed, gray: intermediate shifts. (E) *Pisum sativum* chloroplasts remained untreated (Ch) were incubated with 10 mm PEG‐maleimide under iso‐osmotic conditions (−) or during lysis with SDS (SDS). The reaction was stopped, untreated and iso‐osmotic treated organelles harvested, and all samples subjected to western blotting as in D after acetone precipitation

We probed the protease sensitivity of psToc75‐V in isolated chloroplasts (Fig. 3A,C). Treatment with thermolysin 44, 45 of osmolytically lysed (Fig. 3A, TO), but not of intact chloroplasts (Fig. 3A, T), yielded degradation of psToc75‐V as determined by immunostaining with αToc75‐V antibodies. The efficiency of the treatments was judged by immunostaining of the tripartite Toc159 composed of a cytosolic exposed A and G domain as well as a membrane‐embedded M domain and the β‐barrel protein OEP37. Proteolysis of Toc159 yielded the membrane (M) domain after thermolysin digestion of its cytosolic domains (T), and the M domain was further degraded in osmotically lysed chloroplasts (TO) 37. In turn, OEP37 became only accessible to protease treatment after osmolysis (TO) 37.

The orientation of psToc75‐V was further probed by incubation of intact or osmotically lysed chloroplast with maleimide‐linked polyethylenglycol (5 kDa Peg‐Mal). Peg‐Mal can crosslink to cysteines of surface‐exposed proteins and induces a size shift proportional to the number of modified residues 41. We probed for modification of psToc34, the inner envelope protein psTic110 and psToc75‐V. psToc34 contains a single cysteine in the cytosolically exposed N‐terminal domain 46, 47, while psTic110 contains eleven cysteine residues in its mature domain according to the N terminus suggested by Edman degradation 48, which should be protected by the outer envelope 49. psToc75‐V contains four cysteines in the POTRA domains and one at the seventh predicted ß‐strand facing the opposite membrane surface than the POTRA domains (Fig. 3B,C). Pegylation of the single cysteine of psToc34 yielded a shift of about 10 kDa (Fig. 3D, left). Tic110 became accessible to Peg‐Mal only after lysis of the chloroplasts (Fig. 3D, middle, black arrow). Although a high number of cysteines exist in Tic110, only bands migrating at a lower molecular weight than expected for a full pegylation were observed (Fig. 3D). Pegylation of as many as 11 cysteines in the mature domain might result in size shift exceeding the resolution of the SDS/PAGE. Nevertheless, the reduction of the unmodified Tic110 in the osmotically lysed sample implies an efficient pegylation (Fig. 3D, αTic110, O). Based on the change in the migration, it can be inferred that psToc75‐V was only pegylated at one cysteine before lysis (Fig. 3D, right, −, PEG, gray arrow), while after lysis, at least three cysteines were labeled based on the migration behavior of the upper band (Fig. 3D, right, O, PEG, black arrow).

We evaluated whether the low rate of detection of the pegylated state is the result of intramolecular interactions that block pegylation or whether the pegylation interferes with the immunodetection. To this end, we solubilized the chloroplasts with SDS during the pegylation reaction. This led to 100% pegylation of Toc34 (Fig. 3E, panel left, last lane). The same holds true for Toc75‐V, which became largely pegylated (Fig. 3E, panel right, last lane). However, the intensity of the immunodecorated hyperpegylated Toc75‐V was drastically reduced when compared to the endogenous or monopegylated form (Fig. 3E, panel right). Thus, we conclude that the low detection of the hyperpegylated Toc75‐V after osmolysis (Fig. 3D) is a combination of both, structural constraints and reduced antibodies efficiency. Nevertheless, according to the distribution of the cysteines (Fig. 3B,C), the detection of the hyperpegylated Toc75‐V only after osmolysis (Fig. 3D) in combination with the protease protection (Fig. 3A) suggests an intermembrane space exposure of the POTRA domains.

## Discussion

Ever since the discovery of the 66 kDa orthologue of Toc75‐V in *P. sativum* 11, it was discussed controversially whether this protein contains a cleavable N‐terminal segment 11, 12, 13, 15, 24, 25, 26. The accessibility of the N‐terminal portion of atToc75‐V is not essential for insertion of the protein into the membrane and can be extended by an N‐terminal FLAG tag without interference with translocation (Fig. 1; 13). However, the N terminus of Toc75‐V is processed at a conserved cysteine followed by a consensus sequence (S/C A/T S/T L/I S L T/N/S; Fig. 2), likely after import. This observation is consistent with a recently published observation 27. There are two likely candidates for this process: (a) the stromal processing peptidase in case the N‐terminal region is transferred into the stroma 27; or (b) the plastidic type 1 signal peptidase previously identified to be involved in maturation of atToc75‐III 24. The recently published observation suggests that the cleavage is catalyzed by the stromal processing peptidase 27.

Considering the capacity of β‐barrel proteins to self‐insert into membranes as documented *in vitro*, which is even further accelerated if proteins are present in the membrane 50, 51, the absence of a defined signal could cause targeting to a wrong membrane, insertion with an incorrect topology. However, fusion of POTRA domains to a β‐barrel passenger protein that natively lacks a transit peptide inhibited the membrane insertion of the latter 27. Hence, the N‐terminal portion might contain a targeting signal that ensures, or at least enhances targeting specificity and translocation efficiency. Further, the N terminus might be central for efficient achievement of the correct orientation of Toc75‐V with the POTRA domains exposed to the intermembrane space (Fig. 3; 15). Thus, fusing a tag to the extreme N terminus (Fig. 1; 37) might either inhibit translocation or the tag resides in the stroma after processing. In favor of the last interpretation is our observation that the N‐terminal Flag tag does not inhibit efficient processing (Fig. 1).

The observed orientation of Toc75‐V parallels the one of Sam50, which is involved in the assembly of β‐barrel proteins into the outer membrane of mitochondria 2, 52. Thus, our results (a) support the emerging hypothesis that Toc75‐V contains a cleavable N‐terminal segment 13, 27; (b) support the suggested topology with POTRA domains exposed toward the intermembrane space; and (c) give an explanation for the incorrect topology deduction as a construct with a tag at the extreme N terminus was utilized 37. The authors of the mentioned study 37 based their construct on the proposal by colleagues that Toc75‐V does not contain a cleavable signal 25. The exposure of the POTRA domains to the intermembrane space likely has implications for the proposed function of Toc75‐V in the assembly of plastidic β‐barrel proteins 19, 20, 52, which, however, still needs to be experimentally confirmed.

## Conflicts of interest

The authors declare no conflict of interest.

## Author contribution

ES conceptualized the study. LEG and ES approved the proposal of experiments, designed the experiments, and drafted the manuscript. LEG and NS assessed the performance of molecular, cellular, and biochemical experiments. LEG, SS, and ES carried out bioinformatics analysis. LEG, SS, NS, and ES edited the manuscript.

## Supporting information


**Fig. S1.** psToc75‐V sequence analysis. Total RNA of *Pisum* *sativum *seedlings were used for the preparation of RNA‐seq libraries (GenXPro GmbH, Frankfurt, Germany). The mRNA enrichment was performed via poly(A) selection and purification. Total RNA was strand‐specific sequenced on the HiSeq2500, which resulted in ~ 125 × 106 paired‐end reads of 125 bases. For reference based assembly of the transcriptome BBmap implemented by B. Bushnell (https://sourceforge.net/projects/bbmap/) was used. The *P* *.* *sativum *reads were mapped on the annotated genome of *Medicago truncatula*. The gene models of *M. truncatula *were used as reference for creation of transcripts of *P. sativum*. Differences like insertions, deletions or mismatches at specific positions between *M. truncatula *and *P. sativum *were included to create *P. sativum *specific transcripts. Not covered gaps in exonic regions of *M. truncatula *gene models were used as split point to create mRNA contigs of *P. sativum*. If possible the single exons of the *M. truncatula *gene models were used as starting point to create whole coding sequence (CDS) of *P sativum*. (A) The CDS determined by sequencing and (B) the deduced protein sequence of psToc75‐V.
**Fig. S2.** psToc75‐V sequence alignment. (A) The alignment of the amino acid sequence of Toc75‐V from *Pisum* *sativum,* *Medicago* *truncatula *and *Arabidopsis thaliana* is shown. The black line indicates the POTRA domains and the grey line the ß‐barrel domain. (B) The alignment of the N‐terminal amino acid sequence of Toc75‐V from *P. sativum, M. truncatula, Glycine max, Solanum lycopersicum, Brassica rapa, A. thaliana *and *Chlamydomonas reinhardtii *is shown. Orange bars indicate the region of cleavage and the arrowhead indicates the identified cleavage site. The alignments were created as described in Materials and Methods and visualized with Jalview [53].
**Table S1.** Oligonucleotides.
**Table S2.** Constructs and plasmids used.
**Table S3.** Antibodies used in this study.
