# Supplementary material for: Toc75‐V/OEP80 is processed during translocation into chloroplasts, and the membrane‐embedded form exposes its POTRA domain to the intermembrane space
Source: FEBS Open Bio. 2020 Feb 17;10(3):444–54. doi: 10.1002/2211-5463.12791 (PMC7050246; doi:10.1002/2211-5463.12791)
Supplement: Supplementary file 1 — Fig. S1. psToc75‐V sequence analysis. Total RNA of Pisum sativum seedlings were used for the preparation of RNA‐seq libraries (GenXPro GmbH, Frankfurt, Germany). The mRNA enrichment was performed via poly(A) selection and purification. Total RNA was strand‐specific sequenced on the HiSeq2500, which resulted in ~ 125 × 106 paired‐end reads of 125 bases. For reference based assembly of the transcriptome BBmap implemented by B. Bushnell (https://sourceforge.net/projects/bbmap/) was used. The P . sativum reads were mapped on the annotated genome of Medicago truncatula. The gene models of M. truncatula were used as reference for creation of transcripts of P. sativum. Differences like insertions, deletions or mismatches at specific positions between M. truncatula and P. sativum were included to create P. sativum specific transcripts. Not covered gaps in exonic regions of M. truncatula gene models were used as split point to create mRNA contigs of P. sativum. If possible the single exons of the M. truncatula gene models were used as starting point to create whole coding sequence (CDS) of P sativum. (A) The CDS determined by sequencing and (B) the deduced protein sequence of psToc75‐V. Fig. S2. psToc75‐V sequence alignment. (A) The alignment of the amino acid sequence of Toc75‐V from Pisum sativum, Medicago truncatula and Arabidopsis thaliana is shown. The black line indicates the POTRA domains and the grey line the ß‐barrel domain. (B) The alignment of the N‐terminal amino acid sequence of Toc75‐V from P. sativum, M. truncatula, Glycine max, Solanum lycopersicum, Brassica rapa, A. thaliana and Chlamydomonas reinhardtii is shown. Orange bars indicate the region of cleavage and the arrowhead indicates the identified cleavage site. The alignments were created as described in Materials and Methods and visualized with Jalview [53]. Table S1. Oligonucleotides. Table S2. Constructs and plasmids used. Table S3. Antibodies used in this study. [file FEB4-10-444-s001.pdf]

Supplementary Materials to

Toc75-V/OEP80 is processed during translocation into chloroplasts and exposes the POTRA domains into the intermembrane space

Lucia E. Gross, Nicole Spies, Stefan Simm, and Enrico Schleiff

Supplementary Table S1 Oligonucleotides

Supplementary Table S2 Constructs and plasmids used

Supplementary Table S3 Antibodies used in this study

Supplementary Fig. S1 psToc75-V sequence analysis

Supplementary Fig. S2. psToc75-V sequence alignment

SUPPLEMENTARY TABLES

Supplementary Table S1. Oligonucleotides

Name	Sequence	Enzymes
**Flag basevector**		
pAVA_N-Flag fw	AAGCTTGGTACCGACCATGGACTACAAAGACG	NcoI
pAVA_N-Flag rv	TCTAGATTAACTAGTCGCGGCCGCGGTACCGGATCCGCCACCGGGC CCCTGGAACAGAACTTCC	XbaI
pAVA_C-Flag fw	AATCCATGGGTACCGCGGCCGCATGACTAGTGGTGGCGGATCACTA GAAGTTCTGTTTCAGGG	NcoI
pAVA_C-Flag rv	AATTCTAGACTAACCTTTATCATCGTCG	XbaI
**Flag constructs**		
75V_M53A_PstI	GTAACTCACTAAACCAAGCGCTGCAGTCGCTAAAGAATCGC	Quick change mutagen.
75V_M77A_EcoRI	CAATTTACCGACTCAGGCGCTGAATTCAGTAACCCAGC	
75V_M85A_PvuI	CAGTAACCCAGCTTGCGATCGGGAAGTCTTCTCC	
75V_delLCCA fw	TCTTTGTCTCTAACTAGACCTAACG	
75V_delLCCA_SacI rv	CAGAGGCGAGCTCAGTCCACGGATTG	
**pSP65_psToc75-V**		
Non gibson 75V fw:	GATCGAGCTCCGACGCATAGATTCTTCTAC	SacI
Non gibson 75V rv:	CGATCTGCAGTGCAAATCCTTGAGAGGACG	PstI
psToc75V Gibson FW	GAATACACGGAATTCGAGCTATGCCTCGAAACGACGATATC	Gibson cloning
psToc75V Gibson RV	TACGCCAAGCTTGGGCTGCATTAGTTTCTATAGCCAACCCCAAAG	
**Overexpression constructs**		
pET24c_Toc75VP1-3_his fw	AATCATATGGTGAGTCGAAATGCGGAGGAACG	NdeI
pET24c_Toc75VP1-3_his rv	AATGCGGCCGCTCAGTGATGATGGTGGTGGTGACGCTCGACACAGT TCATGATCAAATCAACC	NotI
pMal_Toc75V_P1-3_His FW	AATGGATCCGTGAGTCGAAATGCGGAGGAACG	BamHI
pMal_Toc75V_P1-3_His RV	AATGCGGCCGCTCAGTGATGATGGTGGTGGTGACGCTCGACACAGT TCATGATCAAATCAACC	NotI

Supplementary Table S2. Constructs and plasmids used

**FLAG constructs**	
pAVA_11N	Sommer and co-worker [37]
pAVA_11C	Sommer and co-worker [37]
pAVA_atToc75-V_11N	Sommer and co-worker [37]
pAVA_atToc75-V 11C	Sommer and co-worker [37]
pAVA_N-Flag	This study
pAVA_C-Flag	This study
pAVA_atToc75-V_N-Flag	This study
pAVA_atToc75-V_C-Flag	This study
pAVA_atToc75-V_M53A	This study
pAVA_atToc75-V_M53A/77A	This study
pAVA_atToc75-V_M53A/77A/85A	This study
pAVA_atToc75-V_N-Flag ΔLCCA	This study
pAVA_atToc75-V_C-Flag ΔLCCA	This study
**Pisum sativum Toc75-V**	
pSP65_psToc75-V CDS	This study
**Overexpression constructs**	
pET24c_Toc75-V_P1-3	This study
pMal_Toc75-V_P1-3_his	This study

**Supplementary Table S3.** Antibodies used in this study		
**atToc75-V_POTRA1-3, animal 1 and 2**	This study	Polyclonal
**αFlag**	Sigma	Monoclonal
**αToc159M**	Sommer and co-worker [37]	Polyclonal
**αToc34G**	Sommer and co-worker [37]	Polyclonal
**αTic110**	Schleiff and co-worker[38]	Polyclonal
**αOEP37**	Schleiff and co-worker [38]	Polyclonal

A psToc75-V CDS

ATGCCTCGAAACGACGATATCTGTTTCGTTTCTTCTTCCATCAAAATCCCTCTTCCTTCC
AAACCCCTAAACACTCCTTTCAAAACCGCACATTCTCACTTCACCAACGCAACTAACTCG
TTTTCTCAACTCATTCACTCGTTCAGAACTCACTCCACCGAGTTAACTCGGTCCGTTCTC
CGGAAATCGCATTCTCTATGTTCGGCGACATTATCACTGACCGACGAGCAAAAAGGCCTG
TCTCTTTCTCCGGCGGAGGAGACTCAATTGAAAACTCGACAGAATGAAGAGAGAGTTTTA
ATCAGTGAAGTATTGGTTCGAAACAAAGACGGCGAAGAACTCGAGAGGAAGGACCTAGAA
GCAGAAGCGGCTCAGGCTCTCAAAGCCTGTAGACCTAATTCGGCTCTCACAGTTCGCGAG
GTGCAAGAGGATGTTCATCGGATTATCAATAGTGGATACTTTTGTTCATGCGTTCCGGTT
GCAGTTGATACGCGTGATGGTATTCGATTGGTATTTCAGGTAGAACCAAATCAAGAGTTC
CAAGGATTGGTATGTGAAGGAGCTAATGTTATTCCGGCCAAGTTTTTAGAGAACTCTTTT
CGAAATGGACATGGGAAAGTTATCAATTTGAGGCGTTTGGATGAGACTATATCTTCTATT
AATGACTGGTATATGGAGCGTGGTCTTTTTGCCATGGTATCAGCTGTTGAGATTCTATCT
GGGGGTATTCTAAAATTACAAGTTTCAGAGGCCGAGGTCAATAATATTTCCATCCGGTTT
CTTGACAGGAAGACGGGTGAGACTACTGTTGGGAAGACAAAACCTGAAACAATACTTCGG
CAAATTACAACCAAGAAGGGGCAGGTCTACAGCATGCATCAGGGGAAAAGAGATGTAGAA
ACTGTATTAACAATGGGGATCATGGAAGACGTTAGCATTATTCCCCAACCCGCAGATACG
GGGAAGGTTGATTTAGTGATGAATGTGGTGGAACGTCCTAGTGGAGGATTTTCTGCTGGT
GGTGGGATATCAAGCGGGATTACAAGTGGTCCACTCAGAGGACTCATTGGAAGCTTTGCG
TATTCTCATAGAAATGTTTTTGGAAGAAACCAGAAACTCAATATTTCCTTAGAGAGGGGC
CAGGTTGACTTAATCGTCCGTGCAAACTACACTGACCCTTGGATCCAAGGAGATGATAAG
CGAACATCTAGAACAATAATGATTCAGAATTCAAGAACGCCCGGAACAATTGTTCATGGT
AACCAGGATGGTAACAGTAACCTGACTATTGGCCGCATCACAGGTGGCATAGAGTTGGGT
CGACCCATTAGGCCTAAGTGGAGCGGAACAGCAGGACTGATTTTCCAGCGTGCCGGAGTC
TGTGACAACAATGGCGTTCCTATCATTAGAGATCGTTACAACAGTCCTCTTACTGCAAGT
GGCAATACCCATGATGATACATTGCTTGCTAAAATTGAGACTGTTTACACTGGTTCTGGT
GAACACGGGTCTTCTATGTTTGTTCTAAACATGGAACAAGGGCTTCCTGTTTTGCCTGAT
TGGTTATCCTTCACTAGGGTGAATGCACGGGGTAGGAAGGGTGTTGAGATTGGTCCTGCT
CGTTTTAATTTAAGTCTCTCTGGGGGCCATGTGGTAGGTAATTTCTCTCCCTATGAAGCT
TTTGCCGTTGGTGGAACAAATAGTGTGAGAGGCTATGAAGAAGGTGGTGTGGGCTCTGGT
CGATCGTATGTTGTTGGCTCCGGAGAAATTTCTTTCCCTGTGAAGCGGCCAGTAGAAGCT
GTCATATTTTCTGACTATGGAACTGATCTTGGATCAGGTTCCACTGTCCTTGGCGACCCT
GCTGGAGCAAGGAATAAGCCTGGAAGCGGATATGGATACGGGTTGGGCATCCGCGTTGAT
TCACCTTTGGGTCCTTTGCGTCTTGAATATGCCTTCAACGACAAGAAAGAGAAGAGGTTT
CACTTTGGGGTTGGCTATAGAAACTAA

B psToc75-V protein sequence

MPRNDDICFVSSSIKIPLPSKPLNTPFKTAHSHFTNATNSFSQLIHSFRTHSTELTRSVL
RKSHSLCSATLSLTDEQKGLSLSPAEETQLKTRQNEERVLISEVLVRNKDGEELERKDLE
AEAAQALKACRPNSALTVREVQEDVHRIINSGYFCSCVPVAVDTRDGIRLVFQVEPNQEF
QGLVCEGANVIPAKFLENSFRNGHGKVINLRRLDETISSINDWYMERGLFAMVSAVEILS
GGILKLQVSEAEVNNISIRFLDRKTGETTVGKTKPETILRQITTKKGQVYSMHQGKRDVE
TVLTMGIMEDVSIIPQPADTGKVDLVMNVVERPSGGFSAGGGISSGITSGPLRGLIGSFA
YSHRNVFGRNQKLNISLERGQVDLIVRANYTDPWIQGDDKRTSRTIMIQNSRTPGTIVHG
NQDGNSNLTIGRITGGIELGRPIRPKWSGTAGLIFQRAGVCDNNGVPIIRDRYNSPLTAS
GNTHDDTLLAKIETVYTGSGEHGSSMFVLNMEQGLPVLPDWLSFTRVNARGRKGVEIGPA
RFNLSLSGGHVVGNFSPYEAFAVGGTNSVRGYEEGGVGSGRSYVVGSGEISFPVKRPVEA
VIFSDYGTDLGSGSTVLGDPAGARNKPGSGYGYGLGIRVDSPLGPLRLEYAFNDKKEKRF
HFGVGYRN

Supplementary Figure S1 psToc75-V sequence analysis. Total RNA of *P. sativum* seedlings were used for the preparation of RNA-seq libraries (GenXPro GmbH, Frankfurt, G). The mRNA enrichment was performed via poly(A) selection and purification. Total RNA was strand-specific sequenced on the HiSeq2500, which resulted in ~125 x10^{6} paired-end reads of 125 bases. For reference based assembly of the transcriptome BBmap implemented by B. Bushnell (https://sourceforge.net/projects/bbmap/) was used. The *P. sativum* reads were mapped on the annotated genome of *Medicago truncatula*. The gene models of *M. truncatula* were used as reference for creation of transcripts of *P. sativum*. Differences like insertions, deletions or mismatches at specific positions between *M. truncatula* and *P. sativum* were included to create *P. sativum* specific transcripts. Not covered gaps in exonic regions of *M. truncatula* gene models were used as split point to create mRNA contigs of *P. sativum*. If possible the single exons of the *M. truncatula* gene models were used as starting point to create whole coding sequence (CDS) of *P. sativum*. **A** The CDS determined by sequencing and **B** the deduced protein sequence of psToc75-V.

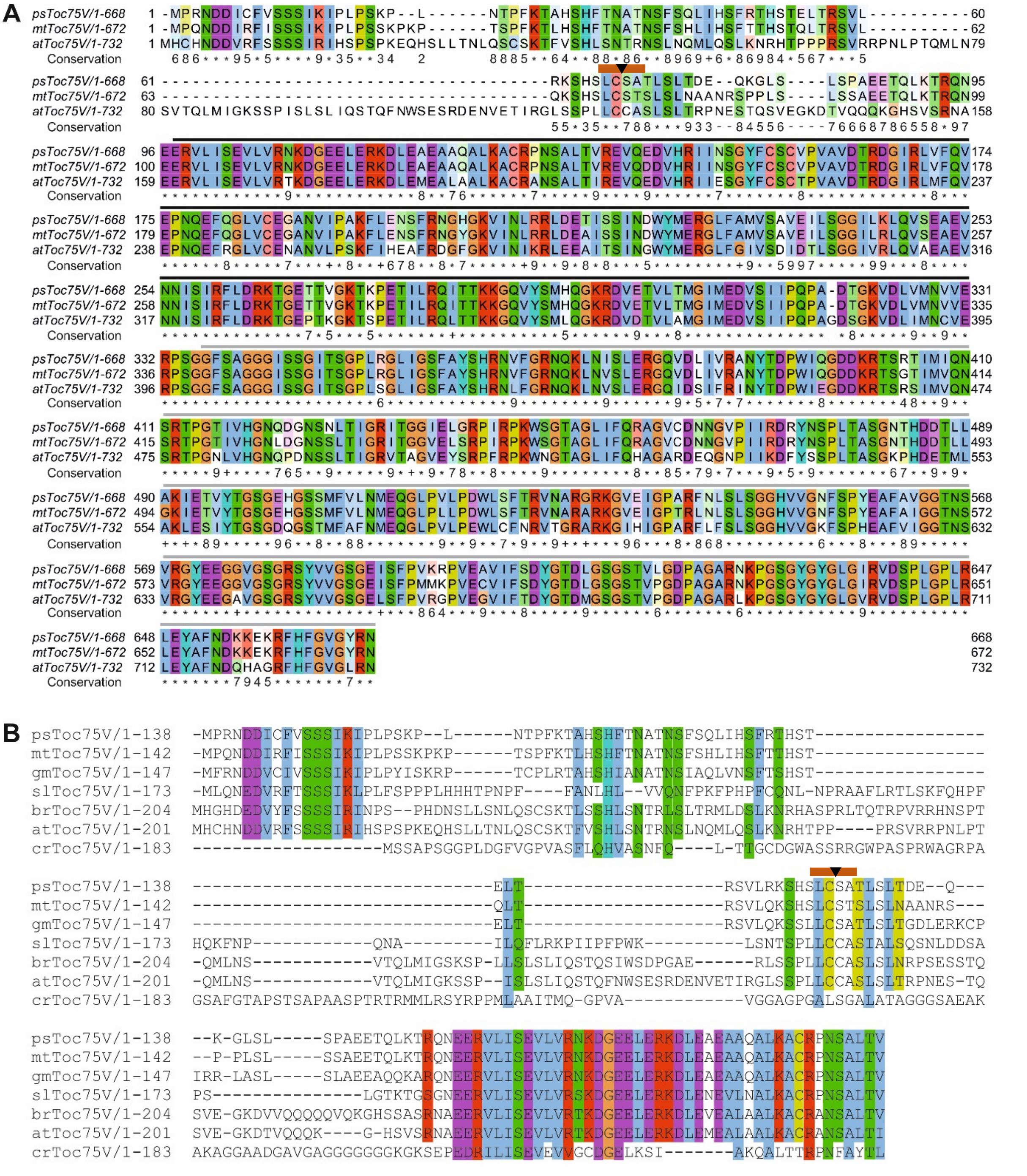

Supplementary Figure S2 Toc75-V sequence alignment. **A** The alignment of the amino acid sequence of Toc75-V from *P. sativum, M. truncatula* and *A. thaliana* is shown. The black line indicates the POTRA domains and the grey line the ß-barrel domain. **B** The alignment of the N-terminal amino acid sequence of Toc75-V from *P. sativum, M. truncatula, Glycine max, Solanum lycopersicum, Brassica rapa, A. thaliana* and *Chlamydomonas reinhardtii* is shown. Orange bars indicate the region of cleavage and the arrowhead indicates the identified cleavage site. The alignments were created as described in Materials and Methods and visualized with Jalview [53].
